# Transepithelial Iontophoresis-Assisted Cross Linking for Progressive Keratoconus: Up to 7 Years of Follow Up

**DOI:** 10.3390/jcm11030678

**Published:** 2022-01-28

**Authors:** Riccardo Vinciguerra, Emanuela F Legrottaglie, Costanza Tredici, Cosimo Mazzotta, Pietro Rosetta, Paolo Vinciguerra

**Affiliations:** 1 Humanitas San Pio X Hospital, 20100 Milan, Italy; 2 The School of Engineering, University of Liverpool, Liverpool L69 3BX, UK; 3 Humanitas Clinical and Research Center—IRCCS, Via Manzoni 56, 20089 Rozzano, MI, Italy; 4 Ophthalmology Unit, Department of Medicine, Surgery and Neurosciences, Post Graduate Ophthalmology School, University of Siena, 53100 Siena, Italy; 5 Ophthalmology Operative Unit, Usl Toscana Sudest, Alta Val d’Elsa Hospital, Campostaggia, 53100 Siena, Italy; 6 Casentino Hospital, 52100 Bibbiena, AR, Italy; 7 Siena Crosslinking Center, Monteriggioni, 53100 Siena, Italy; 8 Department of Biomedical Sciences, Humanitas University, Via Rita Levi Montalcini 4, 20090 Pieve Emanuele, MI, Italy

**Keywords:** keratoconus, cross-linking, iontophoresis, transepithelial

## Abstract

Purpose: To report long-term clinical results of transepithelial cross-linking with iontophoresis (I-CXL) for progressive keratoconus (KC). **Methods:** Nineteen eyes of 19 patients treated with I-CXL for progressive keratoconus were included in this prospective clinical study. Preoperatively and in all available follow ups (6, 12, 24, 36, 48, 60, 72 and 84 months), the following parameters were measured. Corrected distance visual acuity (CDVA), spherical equivalent and cylinder refraction, corneal topography and aberrometry (Costruzione Strumenti Oftalmici (C.S.O.), Florence, Italy), Scheimpflug tomography (OCULUS Optikgeräte GmbH; Wetzlar, Germany). Definition of progression after I-CXL was 2/3 of the following criteria: increase of “A” value, increase of “B” value, decrease of minimal thickness evaluated with the ABCD progression display above 95% confidence interval for post-CXL population when compared to the scan 12 months post-op. **Results:** The mean follow-up time of included patients was 63 months (range 12 to 84 months, 5 patients reached 84 months). The general linear model showed no significant change over time in CDVA, Maximum Keratometry, Thinnest point, and A, B, C values of the Belin Progression Display (*p* > 0.05). Conversely, comatic and high order aberrations decreased significantly over time (both *p* =< 0.001). Five cases (26.31%) showed significant progression after a mean of 55 months (range 36–72) of follow up. **Conclusion:** Our study shows the ability of I-CXL to slow down KC progression in the majority of included patients, improving high order and comatic aberrations. A 26% progression rate was reported.

## 1. Introduction

Keratoconus (KC) is a bilateral ectatic corneal disorder characterized by progressive thinning and steepening; the etiology of the disease is unknown, but there are well-recognized risk factors, such as eye rubbing [1], familiarity and inflammation [2,3,4]. Usually, KC is diagnosed in the adolescence and progresses in the first 3–4 decades of life [5]. Riboflavin-ultraviolet A-induced corneal cross linking (CXL) is a widely accepted procedure to stop KC progression and entails a photo-chemical reaction between ultraviolet light and riboflavin, which aims to stiffen the cornea and stop progression of KC [6]. The first technique was introduced by Dresden University, and it included epithelial removal to allow riboflavin to penetrate corneal stroma (Standard-CXL; S-CXL) [7]. However, epithelial debridement is responsible for most of the side effects of CXL, including postoperative pain, haze, infectious keratitis and decrease of visual acuity [8]. To avoid epithelial removal and its complications, many alternative transepithelial techniques and riboflavin solutions have been proposed [9,10,11,12]. However, most of them did not provide encouraging results when compared to S-CXL [13]. Another solution that was introduced is iontophoresis cross-linking (I-CXL), a procedure in which the drug is applied with an electrode of the same drug charge. A ground electrode, of opposite charge, is located elsewhere on the body to complete the electric circuit. The drug serves as a conductor of the current through the tissue [14]. Many mid-term reports showed the efficacy and safety of I-CXL even when compared to S-CXL [15,16,17,18,19]. A recent long-term study presented 5 years results; however, the published technique differs from the one suggested by the manufacturer (the authors used two continuous cycles of 5 mA/5 min) [20].

For these reasons, the long-term outcomes of this technique have not been clearly demonstrated yet.

Additionally, there is a lack of consensus regarding what should be the definition of progression after CXL [21,22]. Furthermore, the use of a distinct value of anterior curvature as a definition of progression (such as Kmax) is known to be poorly reproducible [23]; some authors suggested to use more than one measurement [24]. Recently, the ABCD progression display system was introduced in the native software of the Pentacam (OCULUS Optikgeräte GmbH; Wetzlar, Germany) to evaluate the progression of KC. The ABCD evaluates average anterior (“A”) and posterior curvature (“B”) values in a 3 mm optical zone centred on the thinnest point of the cornea and in the thinnest pachymetry (“C”) [25]. The “D” value is Snellen CDVA, which is entered manually in the Pentacam software. The software also shows 80 and 95% confidence intervals (CI) for normal and keratoconic population [25]. A recent manuscript introduced the noise of post-crosslinking eyes and created one-sided confidence intervals (CI) for this particular population [26]. The authors suggested to use these CI to assess any significant changes after CXL happening at least 12 months after surgery. 

Based on this report and these confidence intervals, our definition for progression after CXL was at least 2/3 of the following criteria:

- Progression of A parameter more than 95% CI compared to the follow up 12 months after surgery (increase of 0.261 “A” value).
- Progression of B parameter more than 95% CI to the follow up 12 months after surgery (increase 0.208 “B” value).
- Reduction of minimal pachymetry of at least 7 µm compared to the follow up 12 months after surgery (more than 95%CI).

These criteria were already used as a definition of progression in one recently published study that evaluated the 13 years outcome of S-CXL for progressive keratoconus [27]. 

The aim of this study is to report the long-term outcomes of I-CXL in progressive KC with a maximum of 7 years of follow up.

## 2. Materials and Methods

In this, prospective, non-randomized, single centred, interventional study, we included 19 eyes of 19 patients that underwent I-CXL for progressive KC at the Eye Center of the Humanitas Clinical and Research Center (Rozzano, Milan, Italy) between March 2013 and February 2014. 

The inclusion criteria for the treatment of I-CXL were previously published together with the 1 year follow up results [28]. Briefly, progression of keratoconus was defined as a change in maximum keratometry (Kmax) of 1 Dioptre and a reduction of 20 µm in minimal pachymetry. All progressions were confirmed by differential corneal topographies and tomographies. Exclusion criteria were a history of herpetic keratitis, dry eye, severe corneal infection, concomitant ocular or systemic autoimmune disease, diagnosed pregnancy or breastfeeding, the presence of central or paracentral opacities, low compliance and the use of rigid contact lenses for more than 4 weeks before the baseline evaluation.

The study received a formal Institutional Review Board approval from the ethical committee of the Humanitas Clinical and Research Center and was conducted accordingly to the ethical standards set in the 1964 Declaration of Helsinki, as revised in 2013. All patients provided informed consent.

Preoperatively, and in all follow ups (6, 12, 24, 36, 48, 60, 72 and 84 months), the following parameters were measured: corrected distance visual acuity (CDVA), slit lamp biomicroscopy, Goldmann tonometry, dilated fundoscopy, corneal topography and anterior corneal aberrometry for the evaluation of low and high-order aberrations (Costruzione Strumenti Oftalmici (C.S.O.), Florence, Italy) and tomography with Pentacam (OCULUS Optikgeräte GmbH; Wetzlar, Germany) and endothelial biomicroscopy (Konan Specular Microscope, Konan Medical Inc, Hyogo, Japan).

The I-CXL surgery has been already described [28]; it entails the use of riboflavin solution with 0.1% riboflavin, with the addition of two enhancers (Ricrolin +, Sooft, Montegiorgio, FM, Italy). The corneal iontophoresis electrode (I-ON XL, Sooft, Montegiorgio, FM, Italy) generates a constant current of 1 mA (the total dose of 5 mA/5 min is monitored by the generator) for 5 min. UV-A irradiation parameters were: 10 mW (UV-X 2000; IROC Innocross AG, Switzerland) for 9 min for a total energy dose of 5.4 J/cm^2^ [28].

At the end of the surgery, a soft therapeutic contact lens was applied because the high intensity of UV can damage the epithelium for the first days after the procedure. Post-operatively, an ophthalmic gel with 0.3% netilmicin (Xanternet; SIFI SpA, Catania, Italy) was prescribed 4 times a day until no epithelium damage was seen (usually at day 1) and, following removal of contact lens, dexamethasone 21-phosphate 0.15% drops (Etacortilen, Sifi, Lavinaio, Italy) twice daily for 10 days, and 0.15% sodium hyaluronate drops (BluYal, Sooft, Montegiorgio, Italy) 6 times daily for 45 days. Furthermore, all patients received oral amino acid supplements (Aminoftal, Sooft, Montegiorgio, Italy) for 7 days [29]. 

### Statistical Analysis

The statistical analysis was performed with SPSS version 26 (IBM Corp. in Armonk, NY, USA). All data are reported as means ± standard deviation and range. Refractive outcomes were analysed using the methods of Harris and Kaye after transforming the data into Long’s matrix formalism [30,31,32]. 

A generalized linear model (GLM) was used to assess the outcomes and significance. Outcome measures were BCVA, Kmax, Thinnest point, Comatic, Spherical, High Order Aberrations (HOA) and ABC values. Fixed and random factors were age, gender, laterality and follow up time. The level of statistical significance was set at *p* < 0.05. When a significant association was found, Beta coefficients are shown. The beta coefficient displays the degree of change in the outcome variable for every 1-unit of change in the predictor variable.

## 3. Results

Nineteen eyes of nineteen patients fulfilled inclusion criteria. The mean follow-up time of included patients was 63 months (range 12 to 84 months). Mean age was 28.1 ± 6.4 years (range 19–41). Mean preop Kmax was 58.87 ± 3.90 D (range 50.32–64.86), mean preop thinnest point was 435.9 ± 38.25 µm (range 388–523).

### 3.1. Visual Acuity and Refraction Results

CDVA data are expressed in Snellen, while refractive outcomes are shown using the methods of Harris and Kaye after transforming the data into Long’s matrix formalism [23,24,25]. There was no significant association between CDVA and follow up time (*p* = 0.365), and age (*p* = 0.761). However, CDVA was negatively associated with sex (Beta = −0.201, *p* = 0.019) and laterality with right eyes seeing better (Beta = 0.335, *p* < 0.001).

There was no significant change in refraction between baseline and last follow-up available with a mean change of +0.14 sph +1.27 cyl axis 5 and with a standard deviation of ±6.13 sph −4.76 cyl axis 39 (*p* = 0.09).

### 3.2. Topographic and Tomographical Results

Mean baseline and follow-up patients’ parameters are summarized in Table 1.

We found no significant association between Kmax, follow up time (*p* = 0.276) age at treatment (*p* = 0.974) and laterality (*p* = 0.173), while there was significant association with sex (Beta = −1.250, *p* = 0.004). Similarly, the A parameter that evaluates average front curvature in a 3 mm zone around the thinnest point was not significantly associated with follow up time (*p* = 0.179), age at treatment (*p* = 0.852) and laterality (*p* = 0.083), while there was significant association with sex (Beta = −0.469, *p* = 0.021).

We found no significant association between thinnest point (THCT), follow up time (*p* = 0.379), age at treatment (*p* = 0.359), laterality (*p* = 0.330) and gender (*p* = 0.904). C parameters showed a similar trend with no significant association with follow up time (Beta =0.005, *p* = 0.001) but no association with sex (*p* = 0.421), laterality (*p* = 0.601), atopy (*p* = 0.133) and age at treatment (*p* = 0.104).

When considering back surface, the B parameter showed no significant correlation with follow up time (*p* = 0.901), age at treatment (*p* = 0.161) or sex (*p* = 0.052), while there was significant association with laterality (Beta = −0.720, *p* = 0.024).

### 3.3. Aberrometry Results

Spherical aberrations were not significantly associated with follow up time (*p* = 0.393), age at treatment (*p* = 0.727), laterality (*p* = 0.093) nor sex (*p* = 0.168).

**Figure 1:**
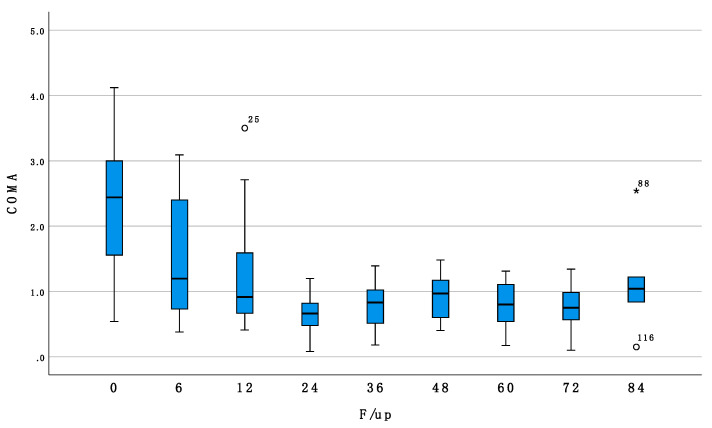
Shows box and whiskers plot of comatic aberrations at baseline and at 6-12-24-36-48-60-72-84 months of follow up.

Conversely, Coma was significantly associated with follow up time, showing significant improvement over time (Beta = −0.015, *p* =< 0.001, Figure 1) but it was not associated with sex (*p* = 0.769), laterality (*p* = 0.230), nor age at treatment (*p* = 0.756).

**Figure 2:**
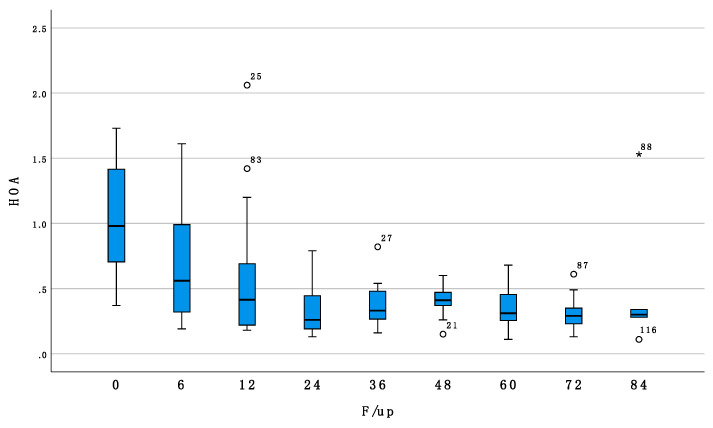
Shows box and whiskers plot of high order aberrations (HOA) at baseline and at 6-12-24-36-48-60-72-84 months of follow up.

Similarly, high order aberrations showed significant improvement over time (Beta = −0.007, *p* =< 0.001, Figure 2) and were significantly associated with age at treatment (Beta = −0.020, *p* = 0.001) but not with sex (*p* = 0.877) nor laterality (*p* = 0.590). 

### 3.4. Adverse Events and Progression Rate

Based on the previously described criteria for failure (progression 2/3 of either “A”, “B” o “C” parameter above 95% confidence intervals for CXL population [26]) five cases (26.31%) showed significant progression after a mean of 55 months (range 36–72) of follow up and, in 4 of them, re-CXL was suggested (with epi off technique). 

One of these five patients required corneal transplant. It should be noted that his preop Kmax was 62.3 and his preop thinnest point was 407 µm. The other four patients had a preop Kmax of 57.9, 56.8, 54.2 and 56.6 D and preop thinnest point of 463, 458, 482 and 405 µm, respectively.

No infections or sterile infiltrates were recorded in this series. As described in our first report [21], one patient developed epithelial oedema, together with pain in the early postoperative period, which healed with medical therapy.

## 4. Discussion

Iontophoresis cross linking (I-CXL) is considered a good alternative to standard epithelium-off CXL (S-CXL) for the treatment of progressive KC, even if it is known to induce a limited flattening when compared to S-CXL [16,17,18]. Preclinical results that assessed the biomechanical effect, riboflavin diffusion and distribution of I-CXL, concur in showing that iontophoresis imbibition is capable of increasing the stromal amount of riboflavin when compared to usual transepithelial [14,33]. Nevertheless, it reached a lower concentration when compared to conventional, epi-off protocol [14,33]. Similarly, stress-strain measurements revealed a significant increase in corneal stiffness after I-CXL when compared to controls, but still lower than S-CXL [34]. There is a lack of evidence in the present literature as to whether this stiffening effect, even if reduced, is enough to halt the ecstatic disease on the long-term.

For this reason, the aim of our study was to present the outcomes of I-CXL with up to 7 years of follow up (mean 63 months).

Our study showed, as main outcome, that I-CXL is able to stop KC progression in the majority of the cases, with a 26% progression rate above the noise level of the instrument [26].

The evaluation and the definition of failure/progression after CXL is of crucial importance. Up to now, most of the reported long-term reports defined failure as:

(1) The event of re-CXL or need for transplant (Raiskup et al.) [35]
(2) Change in Kmax of 1D (Mazzotta et al.) [36]
(3) No definition (Nicula et al.) [37]

The global consensus of keratoconus and ectatic disease stated preoperative progression (before CXL) as a change in at least two of the following parameters where the magnitude of the change exceeds the noise of the testing system [21]:

1. Steepening of the anterior corneal surface
2. Steepening of the posterior corneal surface
3. Thinning and/or an increase in the rate of corneal thickness change from the periphery to the thinnest point.

Based on these recommendations and the ones suggested by a recent Cochrane review [38], we defined our progression criteria as 2/3 of the above criteria using the ABCD system with the recently introduced 95% CI of the CXL population [26] which we already used as definition of progression in our long-term outcomes of Dresden Protocol CXL (S-CXL) [27].

Given that all these patients progressed by an amount greater than the noise level of the measuring system, even if the number of patients included in the study is low, this result should be considered as significant.

It is important to note that if we had based our definition based on a change in 1 D of Kmax, only three patients would have progressed. This last finding is not surprising given the low correlation between Kmax and ABC and the low repeatability of Kmax in KC patients [24,39]. The aim of the “A and B” parameters is to decrease some of this variability by using a more global measurement (curvature taken from a 3 mm optical zone). Additionally, a global measurement reduces the possibility of missing a progression in a KC with an increase of average curvature and a stable maximum curvature.

With the above criteria a 26% progression rate was reported in this series of young-adult patients over 18 years compared to 7.4% of S-CXL using the same parameters [27]. The progression rate in pediatrics in the long-term could be higher according to the faster progression rate and aggressiveness of the disease. This percentage is similar to the data of 23% of keratoconus progression reported by Soeters et al. after transepithelial treatment [40]; however, their report was at 1 year follow up. It is known that I-CXL does not show this progression rate at early follow up times [16,17,18,28].

This evidence agrees with preclinical studies that showed smaller stiffening effect and lower concentration of riboflavin [33,34].

To the authors’ knowledge, this is the first study to report very long-term follow-up of I-CXL since most of the reports showed a maximum of 3 years of follow-up with little or no evidence of progression [15,17,18]. Wu et al. partially concurs with our manuscript and shows a 11% progression rate at 5 years, nevertheless with no clear definition of progression [20].

Furthermore, the latter used a different protocol compared to the one that we presented and that is suggested by the manufacturer. As a matter of fact, instead of the standard iontophoretic current of 5 mA/5 min, they used two continuous cycles of 5 mA/5 min (total 10 mA/10) minutes [20].

It should be noted that our results refer to the standard method of I-CXL with a total energy dose of 5.4 mJ and no pulsed UV-A light. More recent protocols of Iontophoresis CXL with higher total energy dose and pulsed light have shown very promising results [19]; however, more studies will be needed to assess its very long-term efficacy.

The remaining results of our study concur with previous publications and show no significant change overtime in CDVA, Maximum Keratometry, Thinnest point, and A, B, C values of the Belin Progression Display (*p* > 0.05). Conversely, comatic and high order aberrations decreased significantly overtime (both *p* =< 0.001).

The main strengths of our study are the prospective design, the long-term follow-up and the new ABCD-based definition of progression. Conversely, the main limitations are the low number of patients who completed all follow-up visits, which limits the power of the study, the lack of control group (S-CXL) and the single centre design. More long-term studies with a higher number of included patients will be needed to confirm our findings.

In conclusion, long term outcomes of I-CXL show a progression rate of 26% with limited improvement in morphological and functional parameters. This evidence might suggest a change in clinical practice and limit the indication for standard I-CXL to less aggressive/slowly progressive keratoconic patients who are at lower risk or to consider using newer iontophoresis protocols with a higher energy dose.

## Figures and Tables

**Table 1:** Shows details and *p* values of the outcome measures at baseline and at 6-12-24-36-48-60-72-84 months of follow up.

	Baseline	6 Months	12 Months	24 Months	36 Months	48 Months	60 Months	72 Months	84 Months
*N*	19	18	16	15	15	12	12	12	5
Age	28.10 ± 6.42	28.2 ± 6.59	28.8 ± 6.68	28.4 ± 6.40	27.3 ± 6.2	28.1 ± 6.0.298	29.08 ± 7.07	28 ± 5.3	25.8 ± 4.71
CDVA	0.58 ± 0.191	0.75 ± 0.21	0.79 ± 0.25	0.76 ± 0.27	0.75 ± 0.30	0.74 ± 0.19	0.62 ± 0.20	0.64 ± 0.25	0.75 ± 0.25
Kmax	58.87 ± 3.90	58.4 ± 3.4	58.1 ± 3.80	58.7 ± 4.39	59.5 ± 4.31	59.7 ± 3.63	58.9 ± 2.69	59.5 ± 3.86	58.8 ± 5.47
ThCT	435.9 ± 38.25	443.9 ± 37.43	443.1 ± 36.37	439 ± 38	443.2 ± 42.1	450.2 ± 36.46	446.2 ± 38.2	433.5 ± 47.8	421.2 ± 81.1
HOA	1.03 ± 0.402	0.69 ± 0.45	0.59 ± 0.53	0.33 ± 0.18	0.37 ± 0.17	0.396 ± 0.121	0.34 ± 0.16	0.74 ± 0.386	0.512 ± 0.57
COMA	2.31 ± 0.97	1.48 ± 0.91	1.22 ± 0.86	0.66 ± 0.31	0.77 ± 0.36	0.925 ± 0.353	0.81 ± 0.376	0.06 ± 0.08	1.15 ± 0.87
Spher Ab	0.14 ± 0.46	0.28 ± 0.40	0.23 ± 0.41	−0.29 ± 1.30	0.03 ± 0.09	0.26 ± 0.08	0.05 ± 0.064	3.57 ± 1.70	0.76 ± 0.12
A	3.07 ± 1.29	3.34 ± 1.34	3.1 ± 1.4	3.22 ± 1.4	3.38 ± 1,77	3.40 ± 1.24	3.49 ± 1.13	3.57 ± 1.70	3.51 ± 2.34
B	5.58 ± 2.17	5.6 ± 2.27	5.41 ± 2.24	5.35 ± 2.28	5.7 ± 2.06	5.21 ± 1.46	5.47 ± 1.03	5.40 ± 2.11	5.41 ± 2.74
C	2.19 ± 0.80	2.1 ± 0.80	2.1 ± 0.79	1.99 ± 0.85	2.05 ± 0.85	1.94 ± 0.82	1.95 ± 0.75	2.29 ± 0.96	2.18 ± 1.09

CDVA = Corrected Distance Visual Acuity. Kmax = Maximum Keratometry, ThCT = thinnest point, HOA = high order aberrations, Coma = comatic aberrations, Spher Ab = spherical aberrations, “A”, “B”, “C” are the values of the ABCD progression display.

## Data Availability

Original data is not available due to local laws.
